# Public Health Leadership in a VUCA World Environment: Lessons Learned during the Regional Emergency Rollout of SARS-CoV-2 Vaccinations in Heidelberg, Germany, during the COVID-19 Pandemic

**DOI:** 10.3390/vaccines9080887

**Published:** 2021-08-11

**Authors:** Christoph Schulze, Andreas Welker, Anne Kühn, Rainer Schwertz, Benjamin Otto, Laura Moraldo, Udo Dentz, Albertus Arends, Eckhard Welk, Jean-Jacques Wendorff, Hans Koller, Doreen Kuss, Markus Ries

**Affiliations:** 1 Public Health Service Rhein-Neckar District and Heidelberg, 69115 Heidelberg, Germany; c.schulze@rhein-neckar-kreis.de (C.S.); awelker@icloud.com (A.W.); a.kuehn@rhein-neckar-kreis.de (A.K.); r.schwertz@rhein-neckar-kreis.de (R.S.); b.otto2@rhein-neckar-kreis.de (B.O.); l.moraldo@rhein-neckar-kreis.de (L.M.); d.kuss@rhein-neckar-kreis.de (D.K.); 2 Medical Faculty, University of Heidelberg, 69120 Heidelberg, Germany; kontakt@praxis-arends.de; 3 Fire and Disaster Management Agency Rhein-Neckar District, 68526 Ladenburg, Germany; u.dentz@rhein-neckar-kreis.de; 4 CIMIC District Liaison Commands Heidelberg and Rhein-Neckar, 3rd Medical Regiment, German Federal Armed Forces, 89160 Dornstadt, Germany; eckhard-welk@t-online.de; 5 CIMIC District Liaison Command Heidelberg, German Federal Armed Forces, 70374 Stuttgart, Germany; jjwendorff@web.de; 6 Institute of Technology and Innovation Management, Helmut-Schmidt-University, University of the German Federal Armed Forces Hamburg, 22043 Hamburg, Germany; koller@hsu-hh.de; 7 Pediatric Neurology and Metabolic Medicine, Center for Pediatrics and Adolescent Medicine, University Hospital Heidelberg, 69120 Heidelberg, Germany; 8 Center for Virtual Patients, Medical Faculty, University of Heidelberg, 69120 Heidelberg, Germany

**Keywords:** SARS-CoV-2, COVID-19, disaster, disaster management, disaster response, resilience, pandemic, leadership, organizational psychology, learning organization, VUCA world, vaccination, vulnerable populations, public health

## Abstract

The purpose of this work is to share methods used and lessons learned during a comprehensive inter-institutional pandemic disaster response in Heidelberg, Germany, conveying experiences of the regional SARS-CoV-2 vaccination rollout campaign for up to 1,000,000 vaccines in the year 2020. In this volatile, uncertain, complex, and ambiguous (VUCA) environment, the following five strategic elements were pertinent for institutional arrangements so that specific contributions of the various project partners would be available fast without the necessity of extensive negotiations or information exchange: (1) robust mandate, (2) use of established networks, (3) fast onboarding and securing of commitment of project partners, (4) informed planning of supply capacity, and (5) securing the availability of critical items. Planning tools included analyses through a VUCA lens, analyses of stakeholders and their management, possible failures, and management of main risks including mitigation strategies. The method of the present analysis (VUCA factors combined with analyses of possible failures, and management of stakeholders and risks) can theoretically be adjusted to any public health care emergency anywhere across the globe. Lessons learned include ten tactical leadership priorities and ten major pitfalls.

## 1. Introduction

COVID-19 (coronavirus disease 2019) is caused by infection from the novel coronavirus called SARS-CoV-2 (severe acute respiratory syndrome coronavirus 2). By 16 April 2021, 139,214,611 cases and 2,988,960 deaths were reported globally [1]. Vaccination is expected to prevent further spread of SARS-CoV-2. The first SARS-CoV-2 vaccine, based on mRNA technology, received conditional approval in the European Union (EU) on 21 December 2020, and subsequently (as of 16 April 2021), three more vaccines (mRNA and vector-based) were available for use in the EU [2,3,4,5,6]. In order to stimulate rapid rollout of vaccinations, the German Federal government initiated an unprecedented vaccination campaign in November 2020 with clusters of regional vaccination centers working on ambitious timelines to prioritize the protection of at-risk-populations and achieve fast population immunity [7]. 

The purpose of this work is to share methods used and lessons learned during a comprehensive inter-institutional pandemic disaster response in Heidelberg, Germany, conveying experiences of the regional vaccination rollout campaign in the year 2020. The goal is to assess how institutional arrangements can be established or even prepared in advance, so that specific contributions of the various project partners can be available rapidly and at relatively low transaction cost, i.e., without the necessity of extensive negotiations and information exchange [8,9,10]. Five strategic elements were pertinent to streamline institutional arrangements (Table 1). 

## 2. Methods and Theoretical Foundations

This is an empirical narrative-phenomenological autoethnographic review [11,12]. The authorship team was directly involved in this interdisciplinary disaster response mission on a local level and represents the following collaborating institutions and stakeholders: the local public health service, fire and disaster management agency, civil–military collaboration units of the Bundeswehr (Federal Armed Forces of Germany), and the medical faculty of the University of Heidelberg, and for this mission they provided four of the five ingredients identified by Partners in Health as fundamental for strengthening health systems: staff, stuff, spaces, and systems [13]. Two authors (C.S. and A.A.) are medical directors of local vaccination centers with hands-on operational experience. It became clear from very early on in the mission planning that these strategic elements were subjected to phenomena of the VUCA world, i.e., volatility, uncertainty, complexity, and ambiguity [14]. VUCA originated in post-Cold War military thinking after the collapse of the Soviet Union and was later adopted into economic science. It comprises the difficulties and challenges in describing future environmental conditions [15,16]. Elements of the VUCA framework were reviewed by Bennet and Lemoine: Briefly, “volatility” is characterized by frequent and unpredictable changes, “uncertainty” refers to the lack of knowledge as to whether an event will result in a significant change, “complexity” is the presence of an interconnected and convoluted network of information and procedures, and “ambiguity” is defined as the inability to understand cause and effect [17]. VUCA environments are turbulent and tend to be challenging because they can overwhelm leaders and result in inaction due to paralysis, whereas according to Casey “leaders need to ‘see around corners’—to see something significant about the future that others don’t see” and act accordingly in order to be successful [16]. There are various excellent examples for the application of VUCA thinking in the context of medicine or disaster response [18,19,20,21,22,23,24,25,26]. As proposed by Baran and Woznyj, we focused on (1) identifying “our” VUCA in the present mission with (2) the goal to implement agility-enhancing practices while (3) considering obstacles to agility [15]. 

The method of this autoethnographic review is based on a qualitative mixed narrative-phenomenological approach as proposed by Creswell [11,12] as follows: Autoethnographic qualitative data were collected by the core team (C.S., A.W., and M.R.) through videoconferences or face-to-face meetings with multiple in-depth interviews and review of mission protocols. Participants’ stories were transcribed and analyzed for significant statements as the narrow unit of analysis. Individual stories were re-organized into a general VUCA framework. Aggregated stories included a structural description of experiences considering conditions, situations, and context. These aggregated stories and their meaning were shared by the core team with the co-authors for interpretation and feedback [11]. Conflicts were resolved by consensus. The shared phenomenological experience of the team during this disaster response mission was a challenging disaster response mission under high time pressure and wide public visibility. Therefore, stories include a description of “what” and “how” stakeholders experienced. Significant statements in individual stories were horizontalized and primarily grouped into clusters of meaning though a VUCA lens and corresponding planning tools [12]. 

Phenomenological experience included a successful, structured planning process and application of useful planning tools. We will describe each transaction cost-reduction element and link it to corresponding strategic planning tools. This includes an analysis through a VUCA lens of stakeholders, their management, possible failures, and management of main risks that were identified. 

Specifically, an actors’ diagram semantically mapped the convoluted and interconnected environment of actors relevant to the mission. Semantic mapping has the goal to intuitively provide orientation and clarity [27]. Key stakeholder analysis and management analysis provided an insight into commitment to the mission and power of a given player over time [28,29]. This tool allows conscious decisions to be made about the attribution of influence and resources of key stakeholders from a mission perspective. This visual analysis was conducted on two axes, i.e., “supporting change” (commitment) and “impact on success” (power). The situation at the beginning of the project was compared to the current situation and projected by arrows, which show the change of an actor’s impact and support for change. The directions for key players are illustrated with arrows. Here, “impact on success” refers to the power of a particular player for the given project, while “supporting change” refers to the institutional willingness to contribute to the mission. Each player’s position prior to the project was mapped, and the arrows show the degree of empowerment and motivation over time, mainly through leadership by influence.

Risk management includes a possible failure analysis in order to allow the team to think ahead of the curve. This visual tool matches the most important identified risks with their anticipated risk of occurrence and impact on success of the mission. It allows advance planning of mitigation strategies and possible crisis measures in case the pre-identified risk occurs [30]. 

The link between transaction cost reduction elements (i.e., institutional arrangements allowing alignment without the necessity for extensive negotiations) and their corresponding planning and analysis tools is presented in Table 1. The general purpose of the planning tools is to enhance the understanding of the turbulent operation environment. This knowledge can subsequently be applied to combine transaction cost-reducing elements with elements of high agility. The detailed contextual relationship between a specific transaction cost-reduction element and the corresponding planning tool is explained and illustrated in the corresponding thematic paragraph below in the text. Finally, in order to provide a starting point for future mobilizations of public health responses, from local players to regional governments, ten granular tactical leadership priorities and 10 major pitfalls will be presented as lessons learned.

**Table 1:** Five key strategic transaction cost-reduction elements and their linked seven planning tools for the successful rapid scale-up of regional SARS-CoV-2 vaccinations in Heidelberg, Germany, during the disaster response phase of the 2020 COVID-19 pandemic.

Transaction Cost ^#^ Reduction Element	Planning Tool
1. Government mandate	Ambiguity analysis
2. Use of established networks	Complexity analysis
3. Fast onboarding and securing of commitment	Stakeholder [28,29] and possible failure analysis
4. Informed planning of supply capacity	Volatility and uncertainty analysis
5. Secure availability of critical items	Risk management analysis [30]

^#^ Transaction cost is defined as the resources needed to exchange information between potential partners in order to find appropriate partners, to negotiate a cooperation, to monitor the cooperation agreed upon, and to adapt the cooperation to changed requirements [8].

## 3. Transaction Cost-Reduction Measure 1–Government Support

### 3.1. Planning Tool: Ambiguity Analysis

We experienced ambiguity in various fields. There was high pressure for action, while resource availability, in particular personnel and equipment, did not match the ambitious goals. Some initial stretch goals were challenging because there was no follow-up plan yet. Some unaligned public announcement could therefore not be translated into tangible action. There was cognitive dissonance about vaccination among the public. Eligible vaccination candidates refused to be vaccinated, whereas others not yet eligible felt left behind or reacted with anger or threats. While our public health team had experience in vaccinations, the swift startup of a larger vaccination center, in particular with regard to logistic and organizational aspects, was new to us. We started to operate a smaller vaccination center during the migration surge into Germany in 2015, but at that time we dealt with current, established, and widely available products for basic vaccinations as opposed to the new SARS-CoV-2 vaccines for the current project. Disaster relief teams had experience with or were prepared for situations such as flooding, mass causalities, evacuations, or with securing mass events, but not with a pandemic rapidly spreading with exponential growth, or mass vaccination campaigns and their implementation [31,32]. There was ambiguity in long-term personnel planning because there were mixed messages with regard to the future of the center’s operations as well as the precise end of the military personnel support, which created some job insecurity among the staff and may have caused retention issues. Information about vaccine-shipment quantities and timelines was sometimes ambiguous, with implications for operational planning. In general, there was a high commitment of key stakeholders to the mission. 

### 3.2. Mitigation Strategy for Ambiguity: Agility, Based on a Clear Mission

It proved to be very helpful that the intended mission was announced very early by the Federal government in the press and became very widely visible. This gave us a visible mandate for action and reduced the information exchange necessary to convince partners, hence reduced transaction cost. The usual administrative structures in non-catastrophic situations, both civilian and military, can sometimes slow and work in a reactive operational mode. It has been our experience that this lack of drive can create roadblocks and impedes our being ahead of the curve. The Federal Government’s press announcement meant that we could contact potential partners ahead of time to build the mission right from the ground up and save some valuable time, which allowed a precise, consolidated in-depth gap analysis and pro-active planning considering a few possible scenarios. Agile, calculated at-risk planning with proactive options and continuous learning was accepted in an early phase and proved to be beneficial.

## 4. Transaction Cost-Reduction Measure 2–Use of Established Networks

### 4.1. Planning Tool: Complexity Analysis

**Figure 1:**
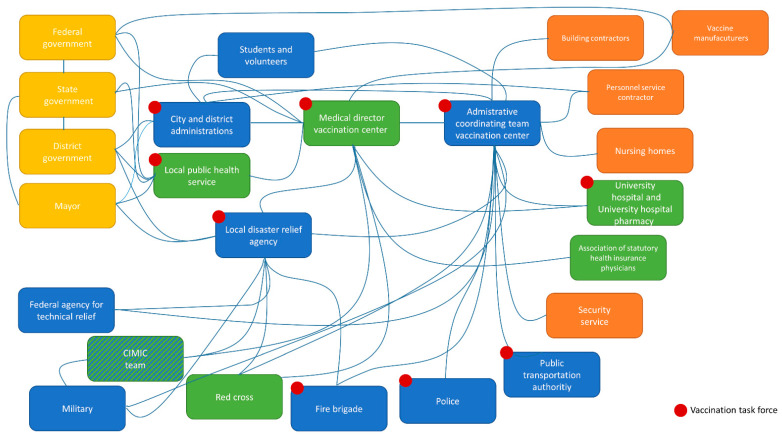
SARS-CoV-2 vaccination disaster response actors’ diagram. There were four groups of players: (1) the political key stakeholders on different layers (yellow), (2) medical services and organizations (green), (3) public or non-commercial organizations (blue), and (4) private or for-profit organizations (orange). Centralized vaccinations were carried out in Heidelberg, Germany, and the surrounding region, i.e., the Rhine-Neckar district, and parts of the Neckar-Odenwald district and Karlsruhe districts covering a population of 750,000 to 1,000,000 potential vaccine recipients. Vaccine centers were supported by mobile teams in order to better reach vulnerable populations. CIMIC = civil–military cooperation.

The environment of actors was complex with many inter-dependencies (Figure 1). Some actors were subjected to their own organization’s political framework that influenced attitudes, actions, and resistance. As there were many players, reaching out to everybody directly and in a timely manner was complex, with potential for misunderstandings and loss of information. Communication in parallel side pathways resulting from this complexity hindered alignment. As several vaccination centers were set up in parallel in neighboring regions and federal states, we found ourselves within a “bidding war” for talent, in particular for medical personnel (physicians, nurses, physician assistants, etc.). IT structures between project modules were not uniform, which rendered communication and documentation complex. Media and societal pressure on politicians leading to misaligned public announcements relating to internal and external key players within the complex conglomerate sometimes caused confusion. Availability of more than one vaccine product complicated handling, procedures, and workflows, thus potentially increasing the risk of mistakes.

### 4.2. Mitigation Strategy for Complexity: Clarity and Concentration by Integrating All Relevant Partners in a Task Force

To enhance clarity and focus, we created an interdisciplinary vaccination task force composed of key stakeholders from medical, administrative, logistic, and security institutions and organizations. We relied on well-established structures and networks, and built relationships to actors that were not yet very familiar to us. Regular informal exchanges with other neighboring vaccination centers on best practices and continuous learning were helpful to increase clarity and to manage common issues such as personnel recruitment. The institutionalized task force as well as building on well-established networks alleviated the information exchange essentially and hence reduced transaction cost during all phases of collaboration. In order to improve transparency, we established a regular monthly staff newsletter. We made the conscious decision to make the newsletter available in paper format and distribute it with wide visibility in the center in order to provide a contrast to the email flood. Some of our staff were seasoned professionals who still preferred paper-based information, and the newsletter could be easily read during breaks, would reach everybody’s attention on site and was considered “more personal” in paper format. In order to be more focused, it was helpful to have constant business partners and fixed coordinators in key sections of the project. Complex, individual handling of the diverse vaccination products with their own specifications respectively required excellent training of the staff, raising awareness of the necessity for precise and concentrated working styles, and a focus on essential aspects. 

## 5. Transaction Cost-Reduction Measure 3–Fast Onboarding of Project Partners and Securing Their Commitment

### 5.1. Planning Tool 3a: Stakeholder Analysis and Management

**Figure 2:**
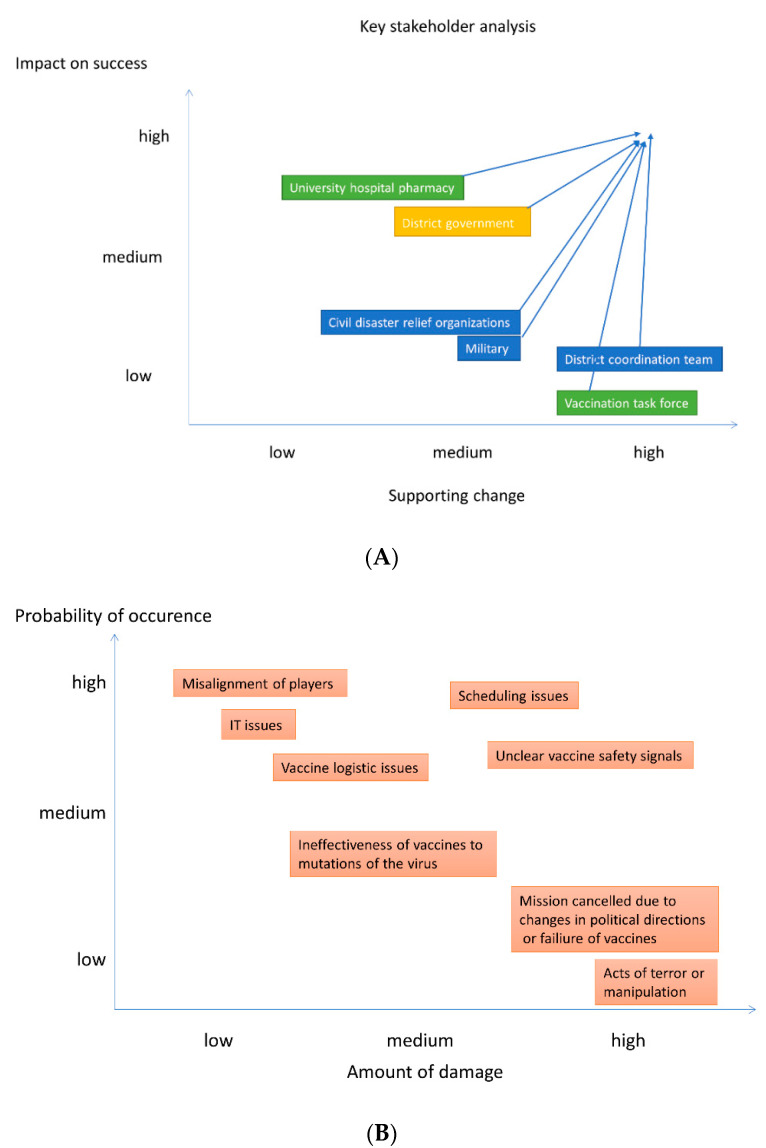
(**A**) (above). Key stakeholder analysis and management. This analysis was conducted on two axes that correspond to power and commitment: “Impact on success” refers to the power of a particular player for the given project, while “supporting change” refers to the institutional will to contribute. The analysis at the beginning of the project was compared to the current situation, with the direction of change in key player management illustrated with arrows. Each player’s position prior to the project was mapped; the arrows show the degree of empowerment and motivation developed over time, mainly through leadership by influence. The vaccine task force set strategic goals; the district coordination team consisted of personnel from a number of offices within the district administration office. The team was made responsible for operational aspects, such as the orders of material and vaccines, as well as the general construction of the vaccination centers and the communication among the different actors. (**B**) (below). Risk management analysis.

In accordance with change-management principles, addressing and integrating key stakeholders was necessary for mission success [28,29]. The stakeholder analysis (Figure 2A) reveals a framework for intended leadership and disaster management across our own organizations and partners. To begin with, it was critical to establish a highly committed vaccine task force and to empower this team quickly. The vaccination task force set high-level strategic goals, then either motivated other powerful institutions to contribute (e.g., the University of Heidelberg hospital pharmacy, disaster relief organizations, and the military) or empowered the district coordination team to become responsible for additional operational aspects. Some stakeholders showed a high will of contribution, while others were restricted by their organization’s structures and policies, which defined their flexibility. Leadership through influence was important. Transaction-cost theory emphasizes that really committed partners are particularly important if their contribution is very specific and strategically important. That is exactly what the stakeholder analysis is trying to find out—in order to reduce transaction cost—from the very beginning.

### 5.2. Planning Tool 3b: Possible Failure Analysis

Important factors of vulnerability could have led to the failure of this project. It was possible that project partners would fail to align. This could result in a situation where cooperation was impossible. 

Business partners could have failed to comprehend the sense of urgency, for example, as a result of being professionally socialized in reactive environments. This could delay swift implementation and decrease public value of the vaccination campaign. Close contact and multi-modal coordinated approaches through a number of different channels were helpful in particular in leveraging relationships on the medical side. As the mission is embedded in a complex structure with organizations subject to their own hierarchies and agendas, it is possible that individuals willing to implement a change “do not find each other” and work in isolation. 

Cross-team transparency and cross-boundary alliance building between institutions is very important. At this point the team was able to build on existing relationships. If senior management of one of the organizations fails to commit to the mission, there is potential for friction within an organization or between organizations. Therefore, it is very important to engage key stakeholders early on. 

A lack of communication of the purpose of this vaccine rollout project (i.e., the vision—to protect the vulnerable and to relieve the healthcare system) in an enthusiastic and convincing way may lead individual team members to get lost in the detail, feel overwhelmed, and become disoriented. This may impede cooperation and operational excellence. Therefore, it is very important to frequently remind team members across organizations about our common key goals and the purpose of the mission. In addition, the integration of powerful agents, for example, government representatives, can foster the engagement of partners. As this is a new type of mission that by necessity moves beyond the comfort zone, it will be very important to anticipate and mitigate resistance in the team early on. 

Failure to demonstrate and build on short-time successes may cause the team and project to lose momentum. Therefore, it is important to render successfully mastered challenges transparent and to praise excellent cooperation for mutual benefit. A positive example is the swift and proactive preparation and launch of the mission focusing on key agents. 

It is possible that SARS-CoV-2 variants develop that become resistant to the available vaccines. This would render the current vaccination program ineffective until an adapted vaccine is available. 

Failure or loss of a vaccine center location or its infrastructure could lead to project failure due to loss of material and spaces. Permanent interruption of the production, distribution, and handling of the vaccine product, including the delivery infrastructure and functioning cooling chain, could lead to failure, as the centers would not have any vaccine to offer to the public. 

If the public is not willing to accept a vaccination offer (e.g., due to lack of trust) the vaccine rollout will not succeed. A transparent and intuitive communication is a key factor for success.

## 6. Transaction Cost-Reduction Measure 4–Informed Planning of Supply Capacities

### 6.1. Planning Tool 4a: Volatility Analysis

Personnel headcount and equipment, in particular IT resources, were locked-in very early in the project, avoiding potential volatility. In contrast, the most important volatility factors included frequent changes to centrally defined priorities and amendments to umbrella guidelines and directives. The initial objective for launch, i.e., administering the first vaccinations, was defined as the moment when “everything was completely ready”. However, changing timelines for vaccine availability required a change into a more agile approach. In the beginning, roles and responsibilities of the centers’ personnel were not clearly defined and were subject to changes depending on the number of vaccinations to be delivered to the public on a daily basis. A critical element for operational planning was the availability of vaccine products. Information about the timelines and quantity of vaccine available changed frequently, which (a) sometimes required immediate action (i.e., within hours) and (b) had a direct impact on personnel motivation that related to the volatility in planning work schedules. There were occasions when critical material was not delivered in time or in the promised quantity, and this led to the need for local supply order initiatives. The selection of a location of the vaccine center initially focused on exhibition halls and on avoiding long-term clashes in usage. Subsequent re-organizations of vaccination administration pathways required adjustments to the interior of the building ranging from minor to more extensive structural changes. Systemic processes such as patient registration and vaccination-rate monitoring evolved over time. 

#### Mitigation Strategy for Volatility: Vision

All involved partners were in full agreement that a fast vaccination of the population was crucial to overcome the pandemic. If partners are convinced of the same, goal information exchange becomes much easier and hence can be done at lower transaction cost. Of central importance is a good understanding of the overall availability of the critical-path element, in our case the vaccine product. Mitigation factors included adaption of operations. In case of a surge of vaccine product availability, staff such as pharmacists can be relocated from other vaccination centers. Potential capacity increases should be considered in the planning phase and include resource buffers. In order to accommodate more vaccine recipients, the center should be prepared to reassign parking spaces and waiting areas. Moving ahead despite risk allowed an increase in vaccination rate: as vaccination products needed to be administered twice within weeks, initially the second dose was kept stored in the pharmacy. Later, the central strategy changed, with 75% of the previously withheld second dose of the product being released, allowing a higher initial vaccination rate in the population assuming, despite the risk, that more product would be received and be available for a second dose within the required time period.

### 6.2. Planning Tool 4b: Uncertainty Analysis

One of the largest uncertainty factors was the accuracy of information about the availability of the vaccination product. In this evolving project, vaccine availability was the critical element for operational planning. Furthermore, there was initial uncertainty about the reconstitution and handling of the newly introduced mRNA-vaccine product. Other uncertainty factors included the fact that there was no single go-to person with a complete situational and directive overview. Roles and responsibilities inside and outside the team as well as in the hierarchies had to be established. Do’s and don’ts were evolving. Information, which was in part contradictory, conflicting, or fragmented, flowed from multiple sources in the hierarchy. There was also uncertainty about the appropriate amount and frequency for information sharing, with attempts to find a balance between the extremes in which important messages were missed on the one hand and there was an unwieldy flood of information on the other.

#### Mitigation Strategy for Uncertainty: Understanding

In order to mitigate against uncertainty, understanding is critical. It was helpful to actively seek available information such as standard operation protocols from other federal states (in our case, North Rhine–Westphalia). In addition, face-to-face conversations with experts or smaller focus teams were helpful in order to achieve alignment in the interpretation of important issues. We were able to use the need for operational synergies with mobile vaccination teams from a neighboring district, i.e., the necessity to agree on common vaccination scheduling, to establish an exchange forum for strategic and scientific questions and to share best practices. Trust was an important factor for information sharing that allowed us to achieve better results in the early phase of the project. Reaching out to contacts with whom we were already networked was valuable, although there were significant time constraints in this dynamically evolving situation.

## 7. Transaction Cost-Reduction Measure 5–Secure Availabilities of Strategic, Specific, and Uncertainty-Subjected Contributions

### 7.1. Planning Tool: Risk Management Analysis

Considering possible reasons for failure as outlined above, eight key risk factors were identified [30]. Each factor was assigned an estimation for (1) probability of occurrence and (2) potential impact on success (Figure 2B). Mitigation strategies are discussed below.

#### 7.1.1. Misalignment of Players

Misunderstandings and differences of opinions among players could lead to conflict that, in turn, could lead to withdrawal or dismissal from the mission. Long working hours could lead to mistakes in complex tasks or trigger unfriendly and uncooperative behavior towards vaccine recipients. Appropriate interface management among the players is very important in order to secure swift flow of information and to avoid friction and confusion.

One potential mitigation strategy against misalignment is to ensure clear, transparent, and multimodal communication with key players to set expectations and detect resistance early. Defining a maximum duration for work shifts can help to prevent mistakes and other employee issues. In our experience, shifts should be limited to 8 h with 16 h shifts only permissible in exceptional situations; these should be limited to two consecutive days. Participation of key players in statewide project meetings was helpful to exchange information about lessons learned for participants facing similar problems. One possible emergency measure is escalation through an individual organization’s hierarchy, which means that knowledge of and relationships to the respective power structures of individual organizations are crucial. An alternative would be to apply peer pressure. An actors’ diagram (Figure 1) can provide orientation required for these measures. 

#### 7.1.2. Scheduling Issues

Vaccination appointments were scheduled by means of a statewide centralized IT system beyond the control of an individual vaccination center. Scheduling issues can occur and result in: open vaccine appointments that cannot be filled; a substantial number of vaccine recipients showing up at a vaccination center who are not eligible according to the current prioritization scheme; or vaccine recipients coming in for a vaccination outside of business hours. This may occur due to an outage of software that was not built to handle a large volume of appointments, programming of false algorithms into the system, or contradictory communication among key institutions. Scheduling issues can also occur if interested vaccine recipients consult a statewide centralized phone hotline and receive contradictory information by undertrained staff. This may shift intense discussions towards to vaccine centers once as ineligible vaccine recipients show up believing that they are eligible to receive their anti-SARS-CoV-2 vaccination.

Mitigation measures include clear communication to the individual, i.e., honest and empathic transparency towards vaccine recipients with ineligible appointments and to the extent possible accommodating situation management. Emergency measures may include timely multichannel communication (e.g., press releases or social media and local webpage postings) or carefully prepared redundancy planning (e.g., to temporarily manage the center’s scheduling process in-house instead of using a state- or nationwide system until critical issues are resolved).

#### 7.1.3. Logistic Issues

Shipping quantities and delivery times for a vaccine product can change abruptly; sometimes we received a short announcement just 14 h in advance. In addition, there were uncertainties about shipments of vaccine accessories such as syringes, needles, etc. Shipment of specialized freezers was delayed. Even the delivery of single-use medical gloves by mail was uncertain at times. 

One possible mitigation strategy is to stockpile critical material in advance. 

It is preferable to work with reliable shipping companies and couriers. However, it is important to avoid creating a single dependency on an individual company, which would lead to issues in the event this company experiences operational problems such as COVID-19 cases among their staff. 

Cooperation with neighboring vaccine centers or hospitals can help mitigate temporary shortages in freezing capacities in the case that the delivery of a specific freezer does not occur on time. In addition, it is possible to store vaccine products for a short time in transport boxes using dry ice. 

Emergency measures to mitigate logistic issues include the procurement of critical parts through alternative suppliers. 

#### 7.1.4. Terror and Manipulation

We have experienced some minor verbal threats by disgruntled vaccine recipients. An incident with a burning garbage container (the cause of which could not be elucidated) next to the vaccine center contributed to an atmosphere of intimidation. Safe and secure storage of vaccine product was a critical priority. 

Mitigation strategies include close cooperation with the police and security companies, installation of a surveillance system including cameras, ensuring key locations are well lit, and securing stockpiles of critical material. It is important to ensure secure transport if the vaccine product is not stocked directly in the vaccine center. Police patrols can deter adverse actions. In addition, building trust through open communication and transparency, as well as though approachability to the extent possible, can be helpful. Trust-building measures such as accessible explanatory videos and social media postings can help de-escalate public tension. IT security and supply of electricity is important, ideally with the availability of redundant systems including emergency power generators. 

Emergency measures include the fast availability of fallback options, such as provisionary tents in order to be able to continue administering the vaccinations (e.g., for situations such as evacuations or bomb threats). Fast police response in case of a critical security incident is important. In addition, the vaccination center should prepare emergency plans in advance. Evacuation exercises can help to familiarize staff with this important safety emergency measure.

#### 7.1.5. Ineffectiveness of Vaccines

If the vaccination campaign is rolled out too slowly, the virus will keep circulating continuously, and the resulting virus mutations could lead to an immunity escape. 

Mitigation factor: a fast implementation of the vaccine campaign makes this scenario less likely. Should there be a stagnation of vaccination due to a lack of eligible vaccine recipients, the vaccine eligibility should be quickly expanded in order to reach a broader population in time. In addition, a more proactive procurement of vaccination product can help to accelerate the campaign rollout.

Emergency measures would include an adaptation of the vaccine product by the manufacturer in order to cover virus variants.

#### 7.1.6. Mission Cancelled

If political directions change, the vaccine campaign could be cancelled. Depending on the timeframe, this could have a serious impact on public health. Should the vaccination campaign be terminated immediately, it is our duty to assess possible negative consequences for the local community and to communicate these risks clearly and in a timely way to appropriate partners. This will avoid unanticipated damage on the reputation of our team.

#### 7.1.7. Vaccine Safety Signals

Uncertain vaccine safety signals can occur that may temporarily stop the vaccination rollout. Mitigation strategies include change and/or cancellation of vaccination appointments with a view to changing to a different vaccination product. Broad and transparent communication through the press and appropriate social media channels, reaching a wide audience, can help maintain trust to avoid rumors and to prevent panic. An emergency measure is a rapid switch to a different vaccination product if immediately available.

#### 7.1.8. IT Issues

It is possible that IT systems become dysfunctional, e.g., after software updates. Mitigation strategies include swift availability of appropriate IT support to the vaccination center and paper backup systems prepared in advance. Emergency measures include a switch to a paper-based documentation system. 

## 8. Limitations

The current analysis is based on a particular sociocultural setting, which has to be considered for a cautious interpretation of the findings and their generalizability: Heidelberg comprises a southwestern German university city with ~160,000 inhabitants and its rural surroundings in an affluent economic area. Legislation for this particular public healthcare setting and the corresponding public–private and civil–military institutional arrangements are governed by the legal framework of the European Union, the Federal Republic of Germany, the Federal State of Baden-Württemberg, and local entities, which may not necessarily be transferable into different frameworks. The aspects of work relating to human behavior, motivation, and interaction per se, however, may be relatively universal and constant across space and time.

## 9. Lessons Learned and Conclusions

A VUCA environment can be challenging in public health emergency situations as illustrated by the current example of a regional vaccine rollout campaign during the COVID-19 pandemic. Five core resilience factors to overcome VUCA friction were identified: (1) a robust mandate, (2) the use of established networks, (3) fast onboarding and securing commitment of project partners, (4) informed planning of supply capacity, and (5) securing the availability of critical items. The method of the present analysis (VUCA factors combined with analyses of possible failures, and management of stakeholders and risks) can theoretically be adjusted to any public health care emergency anywhere across the globe. Mindful leadership in a learning organization is of essence in comparable VUCA scenarios [24,33]. We are therefore proposing 10 top leadership priorities to consider and 10 major pitfalls to avoid that may inform and facilitate the operationalization of comparable endeavors in similar situations in the future (Table 2). 

## Figures and Tables

**Table 2:** Lessons learned from the set-up and operationalization of a regional vaccination center during the COVID-19 disaster response vaccination rollout campaign: top 10 leadership priorities and 10 major pitfalls.

Top 10 Priorities	10 Major Pitfalls
1. Create a task force with all relevant partners so all information can be shared, also create smaller expert groups	1. Change standard operation procedures frequently
2. Involve actual end-users of the centers in the planning phase	2. Set unrealistic goals
3. Start giving information to the operators of the vaccination centers as fast as possible, through clear and easy means of communication	3. Open vaccination centers prematurely
4. Start an early information campaign towards the general public to keep everyone involved	4. Start acting before core planning is finalized
5. Listen to the staff members and respect users	5. Open too many vaccination centers at once in the starting phase if vaccine supply is limited
6. Have a functioning system and appropriate standard operation procedures that can change in response to lessons learned	6. Delay the second step of the rollout phase, i.e., shifting vaccinations from center to doctors’ offices
7. Adapt quickly to new situations	7. Create a whole new system instead of involving and building on already existing structures
8. Have common standards that apply to vaccination centers in the whole country to avoid “vaccination tourism“	8. Work with the cheapest contractor without considering how experienced that contractor is
9. Set aside any prior conflicts with organizations or institutions, work together as a team for the best results possible	9. Enforce standard operation procedures upon vaccination centers without respect to their local conditions
10. Create ways for vaccination centers to communicate and/or visit each other to learn from each other and to avoid unnecessary mistakes	10. Stick to existing hierarchy structures to control everything–even if that causes delays

## Data Availability

All data relevant to this work are included in the article.
